# Pathological Examination of Radiologically Fused Interbody Tissue Five Years After Anterior Cervical Discectomy and Fusion Using the Titanium Cage System: A Report of Two Cases

**DOI:** 10.7759/cureus.28059

**Published:** 2022-08-16

**Authors:** Yoshinori Maki, Toshinari Kawasaki, Kota Nakajima, Motohiro Takayama

**Affiliations:** 1 Neurosurgery, Hikone Chuo Hospital, Hikone, JPN; 2 Rehabilitation, Hikari Hospital, Otsu, JPN; 3 Department of Neurosurgery, Otsu City Hospital, Otsu, JPN

**Keywords:** radiculopathy, titanium cage, pathology, cervical spondylosis, bony fusion, anterior cervical discectomy and fusion

## Abstract

This case report aimed to identify potential relationships between pathological and radiological assessments of bony fusion after anterior cervical discectomy and fusion (ACDF). ACDF can resolve neurological symptoms related to cervical spondylosis, such as myelopathy and radiculopathy. Intervertebral bony fusion is a key outcome for successful ACDF, often assessed on radiography and computed tomography (CT) images. However, the pathological findings of tissues demonstrating bony fusion after ACDF have not been well studied.

This report presents the cases of two female patients, aged 62 and 40 years, who underwent additional ACDFs for recurrent cervical radiculopathy. Findings from CT imaging identified intervertebral calcification in the titanium spacers placed in the first ACDF. In both cases, recurrent compression of nerve roots was observed radiologically. Cervical nerve root block identified habitual symptoms related to recurrent radiculopathy. To resolve the clinical symptoms, additional ACDFs were performed in two cases. In the second ACDF, the titanium cases from the prior ACDF were removed. Histopathological examination of the tissues from the removed cages revealed growth of cartilage tissue.

This is the first report concerning the histopathological evaluation of the tissue in titanium spacers placed in ACDF. Completion of intervertebral calcification in titanium spacers placed in ACDF may not signify completion of intervertebral bony fusion after ACDF.

## Introduction

Cervical spondylosis can result from the degenerated disc. The degenerated cervical disc can bulge medially and laterally, narrowing the foraminal and spinal canal. As a result, radiculopathy and myelopathy can occur and reduce the activities of daily living of patients with cervical spondylosis [1]. To resolve clinical symptoms related to cervical spondylosis, anterior cervical spine surgery can be selected for those cases in which cervical spondylosis involves maximally three disc levels or in which vertebral fusion or vertebral body removal is necessary [2]. Anterior cervical discectomy and fixation (ACDF) is an established method for treating symptoms related to cervical spondylosis [3-6]. ACDF is designed to yield complete bone fusion of operating segments. To complete vertebral fusion in ACDF, an autograft or artificial bone or a metal cage is usually placed in the intervertebral space [2]. Vertebral fusion after ACDF is usually evaluated on postoperative X-ray and computed tomography (CT) images [7]. However, little is known regarding the correlation of pathological and radiological findings of lesions treated with ACDF. Herein, we report two cases, in which pathological findings of the tissue in titanium spacers were examined after ACDF had been conducted more than five years ago.

## Case presentation

Case 1

**Figure 1:**
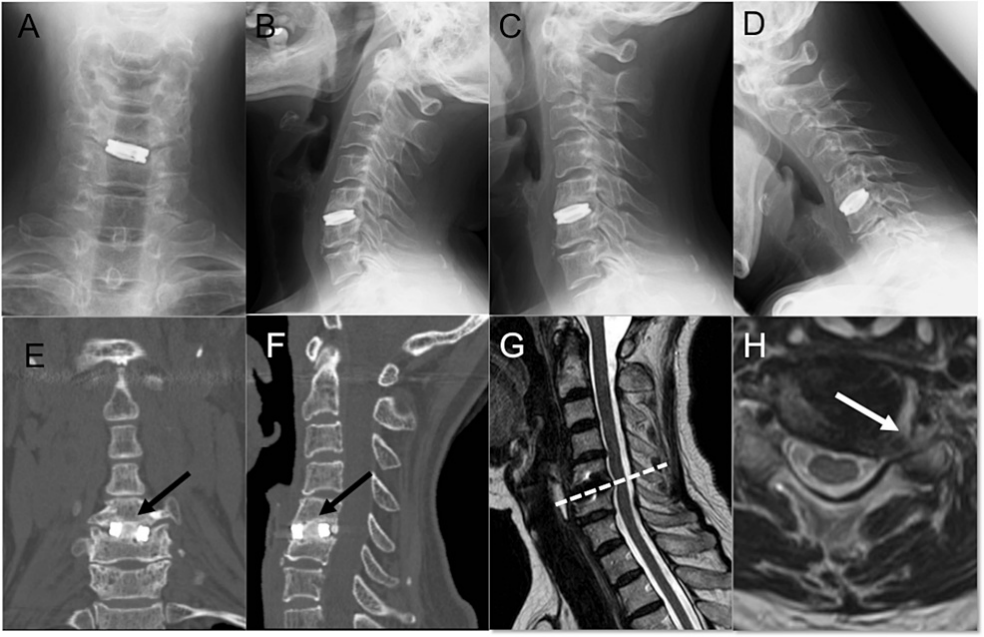
Preoperative radiological findings of Case 1 A titanium spacer was observed at the C5/C6 level. Instability was observed at the C4/C5 level in the flexed position. Instability at the C5/C6 level was not apparent (A: anteroposterior X-ray image, B-D: lateral X-ray images). Calcification in the titanium cage was considered in computed tomography images. The signal intensity of the calcification in the titanium cage was different from that in the vertebral bones (black arrows) (E: axial image, F: sagittal image). The left nerve root of C6 was compressed owing to the presence of osteophytes (G and H: magnetic resonance images).

A 62-year-old woman underwent ACDF for cervical herniation of the C5/6 vertebrae seven years previously. A titanium spacer was placed in the intervertebral space of C5/6 following the removal of the intervertebral disc at C5/6 during the initial ACDF. The endplates of the vertebral bodies were preserved. An artificial bone or autograft was not placed in the titanium spacer (CeSpace XP, AESCLUP®; B Braun, Melsungen, Germany) during the first ACDF. The patient visited Otsu City Hospital complaining of pain in the left lateral upper extremity radiating to the first, second, and third fingers. Neurological examination revealed a motor weakness of the left extensor carpi muscle (manual motor testing, 4/5) The left triceps brachii muscle tendon reflex attenuated. The patient was also examined using radiography and CT. X-ray imaging of the flexion position revealed slight cervical instability at C4/5 (Figures 1A-1D). Calcification was observed in the titanium spacer at C5/6; however, the signal intensity of the calcification was different from that of the adjacent vertebral bodies (Figure 1E, F). Furthermore, magnetic resonance imaging (MRI) identified canal stenosis of the left C6 vertebral foramen (Figures 1G-1H). The habitual pain was confirmed with a nerve root block targeting the left C6 nerve.

**Figure 2:**
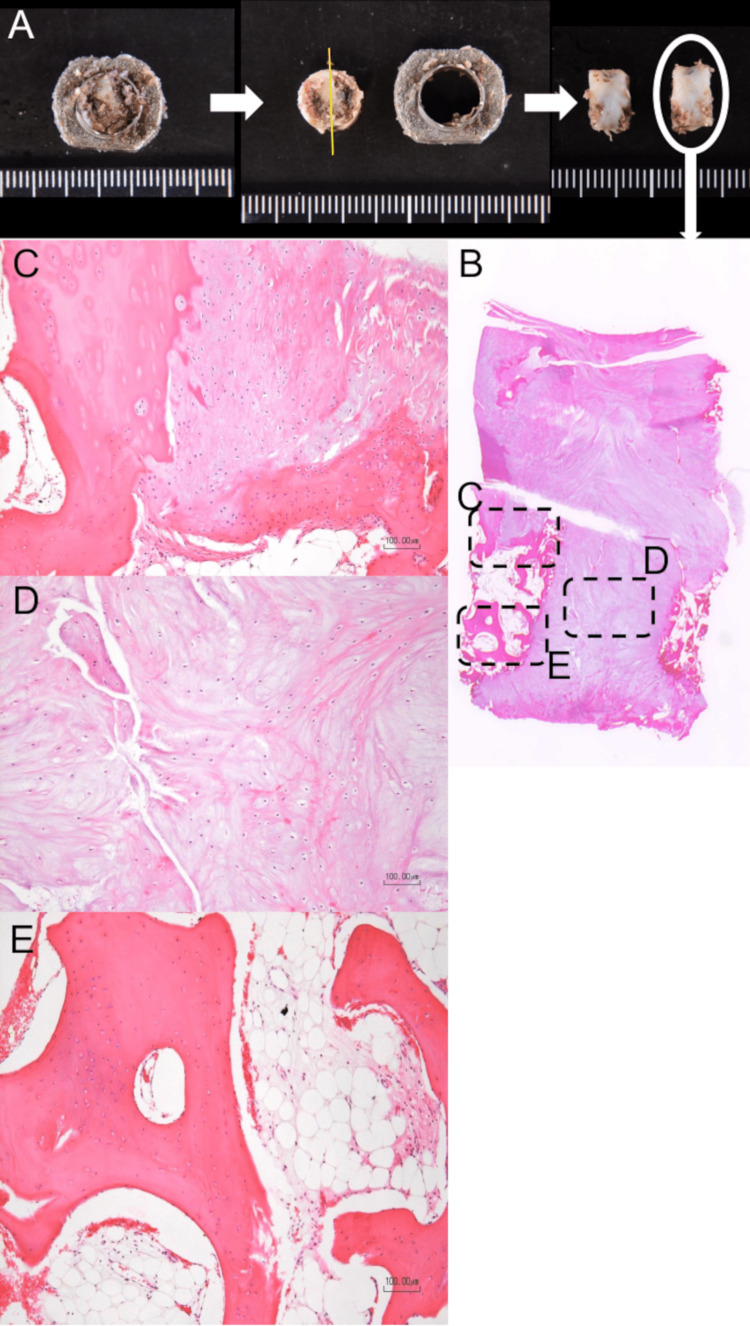
Pathological findings of Case 1 The removed ring titanium spacer was examined pathologically (A). The tissue in the titanium spacer consisted mostly of fibrocartilage. Hyaline cartilage and bone tissue were partially identified (B-E: hematoxylin-eosin staining of the specimen. C: hyaline cartilage tissue, D: fibrocartilage tissue, E: bony tissue).

The titanium cage placed in the first ACDF was easily removed, followed by an additional ACDF of the C5/C6 for symptom resolution, utilizing a titanium plate (VENTURE^TM^, Medtronic, Dublin, Ireland) and cylindrical titanium interbody cage (AFFINITY® Anterior Cervical Cage System, Medtronic) filled with artificial bone. Histopathological evaluation of the tissue from the removed titanium spacer indicated a majority presence of fibrocartilage tissue, with some evidence of hyaline cartilage and bony tissues (Figure 2).

Case 2

**Figure 3:**
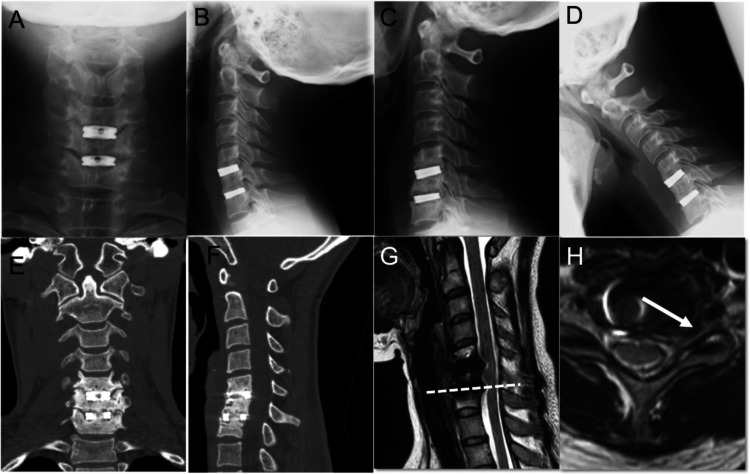
Preoperative radiological findings of Case 2 Titanium spacers were observed at the C5/C6 and C6/C7 levels. Cervical instability was not observed (A: anteroposterior X-ray image, B-D: lateral X-ray images). Calcification was observed in the titanium cages. Complete bony fusion was not achieved on computed tomography (E: axial image, F: sagittal image). The left nerve root of C7 was compressed owing to the presence of osteophytes (G and H: magnetic resonance images).

A 40-year-old woman underwent ACDF for cervical herniation at C5/6 and C6/7 five years previously. In this case, the titanium spacers (AESCLUP® CeSpace XP) were placed without artificial bone or autograft implantation and the endplates of the vertebral bodies were left intact. She experienced pain in her neck and left lateral upper extremity radiating to the first, second, and third fingers. The symptom appeared after she had a traffic accident. Motor weakness was observed in the extensor digitorum and flexor carpi muscles (manual motor testing: 4/5). The left brachioradial muscle tendon reflex was attenuated. Cervical instability was not evident in the X-ray images (Figures 3A-3D). There were minor indications of calcification in the titanium spacer at the level of C6/7; however, bony fusion was incomplete (Figures 3E-3F). The left C7 nerve root was compressed by an osteophyte resulting in recurrent pain in her neck and lateral upper extremities (Figures 3G-3H). The habitual pain was confirmed with a nerve root block targeting the left C7 nerve.

**Figure 4:**
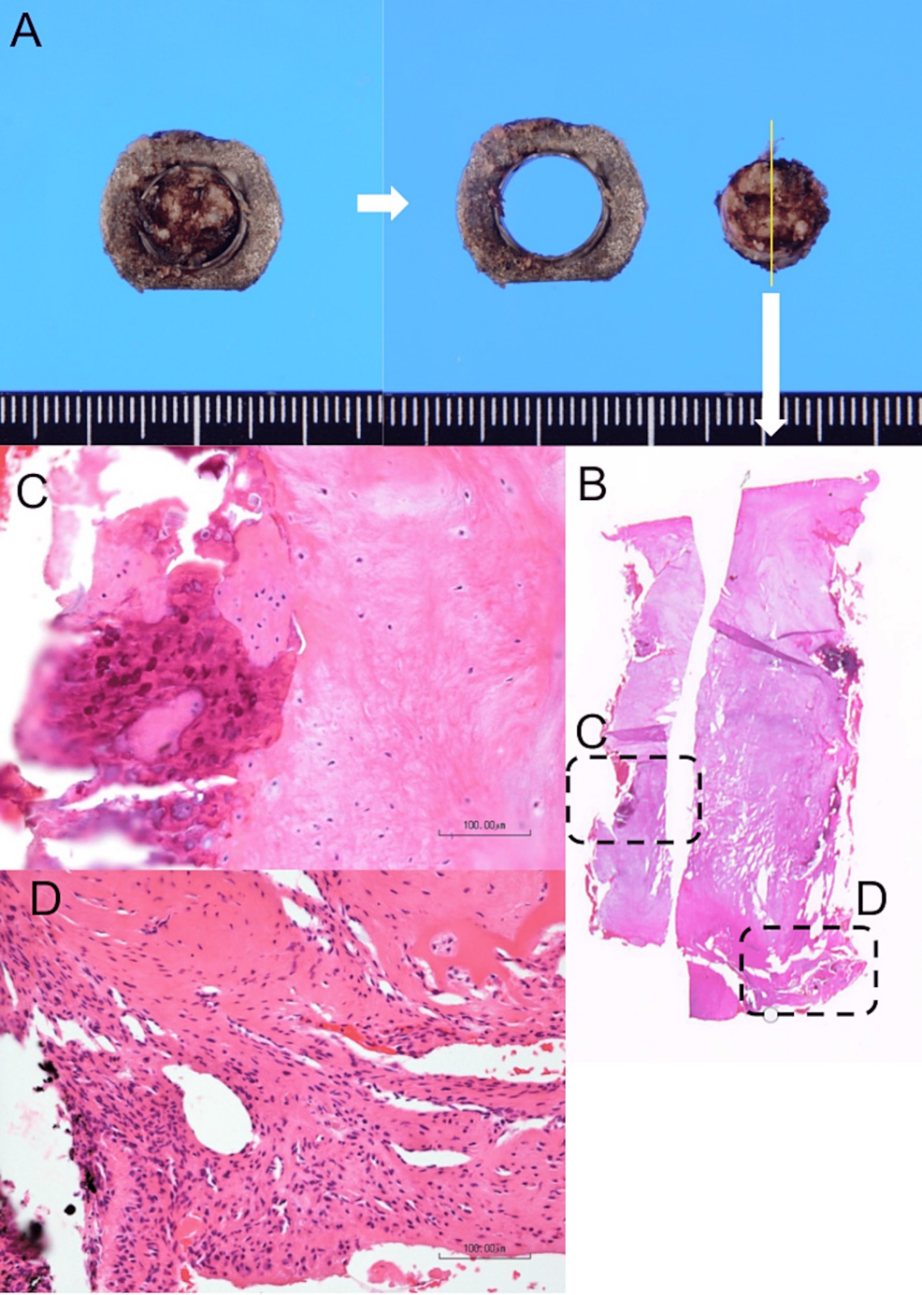
Pathological findings of Case 2 The removed ring titanium spacer was examined pathologically (A). The tissue in the titanium spacer consisted mostly of fibrocartilage. Hyaline cartilage, bone tissue, and synovial tissue were partially identified (B-D: hematoxylin-eosin staining of the specimen. C: hyaline cartilage, fibrocartilage, and bony tissues; D: granulation tissue).

To resolve the symptoms, a second ACDF of C6/C7 utilizing the same devices as in Case 1 was performed following the removal of a titanium cage. Cartilage tissue was also observed in this case (Figure 4).

## Discussion

Here, we describe two cases in which the tissue in the titanium spacers placed in the preceding ACDF was examined histopathologically. Although calcification was identified in the titanium spacers on the CT images, the tissue in the titanium spacers mainly consisted of cartilage tissue.

There are several operative methods in ACDF: using autograft, allograft, or a metal cage [2]. Especially, ACDF using a metal cage can reduce complications related to the iliac bone harvest. ACDF using metal cages can contribute to the high bony fusion rate and low complication rate [2]. However, bony fusion following ACDF has been evaluated with follow-up radiological images [2]. Bony fusion after ACDF based on pathological findings is not described.

The titanium spacers used in ACDF are placed in the intervertebral space, which is superiorly and inferiorly margined by endplates. The endplates are composed of two layers: the outer bony endplate and the inner cartilage endplate [8]. Titanium spacers attach to the surface of the cartilage endplates when the endplates are not curetted with ACDF. In our cases, the endplates were left intact in the first ACDF, and titanium cages were placed in the intervertebral space without a bony graft. The cartilage tissue in the titanium spacers could have resulted from the growth of cartilage tissue from the inner cartilage endplates. In addition, vertebral degenerative diseases are associated with degenerative cartilage endplates [9]. Cartilage cell proliferation and degenerated fibrocartilaginous stroma are often observed in herniated lumbar intervertebral discs [10]. The underlying cervical degeneration from the ACDF presents another potential contributor to the growth of fibrocartilage tissue in the titanium spacers. Bone tissue and hyaline cartilage were partially detected in both cases. The bony tissue could have been derived from the exposed outer bony endplates that may have been scratched by the titanium spacers. Interestingly, hyaline cartilage has not been described in degenerative intervertebral discs [11]. Further studies are needed to determine whether hyaline cartilage is a specific feature of ACDF. As the tissue in the titanium spacers consisted mostly of cartilage, it seems reasonable to suggest that bone grafts in the titanium spacers should be warranted to facilitate bony fusion. This topic should also be addressed in the future, as it lacked the histopathological findings of tissue in titanium spacers in a long-term follow-up.

## Conclusions

In this case report, we presented unique histopathological findings from the tissue in ACDF titanium cages during long-term follow-up assessments. The tissue in the titanium cages utilized in ACDF consisted mostly of fibrocartilage tissue with minor indications of bone and hyaline cartilage. Bony fusion in the tissue in ACDF cages in our cases was incomplete probably because any autograft or artificial bone was not placed in ACDF titanium cages. Autograft or artificial bone should be placed in ACDF titanium cages for postoperative bony fusion. However, as the postoperative pathological findings of ACDF titanium cages filled with autograft or artificial bone are lacking in the literature, further studies are warranted to evaluate the relationship between the pathological and radiological findings of the tissue in titanium cages of ACDF using autograft or artificial bone. Understanding this relationship may yield a better understanding of incomplete fusion outcomes from ACDF that require additional procedural interventions.
